# Non-destructive assessment of tissue engineered cartilage maturity using visible and near infrared spectroscopy combined with machine learning

**DOI:** 10.1016/j.bios.2025.117587

**Published:** 2025-05-14

**Authors:** Omar Anwar Elkadi, Florencia Abinzano, Ervin Nippolainen, Ona Bach González, Juha Töyräs, Riccardo Levato, Jos Malda, Isaac O. Afara

**Affiliations:** a Department of Technical Physics, University of Eastern Finland, Kuopio, Finland; b Department of Orthopedics, University Medical Center Utrecht, Utrecht University, Utrecht, the Netherlands; c School of Electrical Engineering and Computer Science, The University of Queensland, Brisbane, Australia; d Science Service Center, Kuopio University Hospital, Kuopio, Finland; e Department of Clinical Sciences, Faculty of Veterinary Medicine, Utrecht University, Utrecht, the Netherlands

**Keywords:** Fiber optics, Continuous monitoring, Optical spectroscopy, Multimodal spectroscopy

## Abstract

Tissue engineering is a promising approach to address the unmet clinical need for treating cartilage damage. Monitoring the characteristics of tissue-engineered cartilage constructs (TECs) during culture is critical for optimizing culture conditions and assessing TEC development. An important characteristic of TEC development is the ratio of glycosaminoglycans (GAGs) to DNA. However, current methods for quantifying GAGs and DNA are destructive, making them impractical for longitudinal monitoring. This study investigates the potential of optical spectroscopy in the visible and near-infrared (NIR) ranges coupled with machine learning (ML) for non-destructive prediction of GAGs and DNA in TECs. Six types of TECs were incubated for 7 or 28 days (n = 6 × 2 × 3). Spectral data were acquired from the constructs in the NIR and visible-short-NIR (VIS-SNIR) ranges using a fiber-optic probe and a multichannel spectrometer, followed by biochemical quantification of GAGs and DNA. ML models were developed for predicting the GAGs and DNA from the spectra and evaluated by leave-one-sample-out cross-validation after determining the optimal spectral range, preprocessing method, and ML algorithm. The performance of the models for predicting the construct’s relative maturity (based on culture duration) using the predicted GAGs/DNA ratio was also evaluated. A NIR-based model was optimal for DNA content prediction (*R*^2^ = 0.81), while a VIS-SNIR-based model was optimal for GAGs prediction (*R*^2^ = 0.79). The pre-dicted GAGs/DNA ratios were 100 % accurate in distinguishing immature TECs from more mature ones. Thus, optical spectroscopy combined with ML can enable non-destructive prediction of the maturity of TECs via predicting their DNA and GAG contents.

## 1 Introduction

Articular cartilage is a connective tissue composed of specialized cells called chondrocytes, which are embedded within an extracellular matrix (ECM) produced by the same cells during an earlier stage in their development. This ECM primarily comprises water, type II collagen, and proteoglycans. These components provide articular cartilage with unique mechanical properties, enabling its function in the joints as a low-friction shock absorber (Griffith et al., 2021; Hollander et al., 2010). As a hypocellular tissue with a poor blood supply, cartilage has limited self-repair capacity. Thus, once damaged, cartilage degeneration often progresses over time, eventually leading to osteoarthritis (Zelinka et al., 2022; Zylinska et al., 2021).

Tissue engineering (TE) offers a promising approach for replacing damaged cartilage. A common strategy for cartilage TE involves seeding chondrogenic cells into a suitable scaffold and culturing the construct under chondrogenic conditions *in vitro* until it reaches sufficient maturity to replace damaged cartilage (Castro et al., 2022; Levato et al., 2017). As an indicator of construct readiness for potential clinical application, maturity is also a key metric for evaluating TE strategies, including testing novel biomaterials and optimizing growth factor protocols (Jin et al., 2011; Levato et al., 2017).

Conventionally, maturity is assessed using biochemical methods that estimate the ECM produced per cell, with the glycosaminoglycans (GAGs; main components of proteoglycans) and DNA contents commonly measured as indicators of the ECM and cell contents, respectively (Power et al., 2020). However, these methods are destructive, making them impractical for longitudinal monitoring of tissue growth, which is necessary due to the heterogeneity of the cultured tissue (Kandel et al., 2020; Power et al., 2020). For instance, donor-to-donor variations may lead to differences in the culture time required to achieve a sufficiently mature cartilaginous structure (Kim et al., 2018). Moreover, culturing tissues from the same cell source under the same conditions can produce tissues with varying properties. Thus, constructs grown with the same culture parameters (even within the same batch) can require different culture time to reach maturity (Castro et al., 2022; Kandel et al., 2020). Therefore, non-destructive methods are needed to assess the maturity of the construct intended for patient treatment or further research, and to monitor its growth during culture to determine the optimal harvest time and improve resource efficiency.

Optical spectroscopy can non-destructively provide chemical and morphological information about broad types of samples, including biologicals with complex structures, such as tissue-engineered cartilage (TEC) (Afara et al., 2021; Kandel et al., 2020; McCorry et al., 2023). This information is based on the wavelength-specific interactions of electromagnetic radiation (EMR) with the samples at the molecular level. For instance, EMR in the UV/Visible region (200–700 nm) can induce wavelength-dependent electronic transition when interacting with the chromophores in the samples (Rocha et al., 2018). Similarly, EMR in the near-infrared (NIR) range (700–2500 nm) can induce wavelength-specific molecular overtone and combination vibrations of bonds (Afara et al., 2021). These interactions produce specific absorption patterns in the spectra of the samples, providing chemical information about their composition. Moreover, scattering of the EMR, for instance, can provide morphological information about the sample components. This scattering also depends on the wavelength in addition to the size and shape of the analyzed particles (Afara et al., 2021; Drieschner et al., 2020; Vaidyanathan et al., 2003). However, due to the broad and overlapping bands in these spectral ranges, chemometric methods, which more recently include machine learning (ML), are required to utilize these spectroscopic methods (Drieschner et al., 2020; McCorry et al., 2023; Zhang et al., 2022).

As the same light source can provide both spectral ranges, visible and NIR spectroscopy (NIRS) can be utilized simultaneously, providing complementary information, which can be particularly valuable when the target of interest depends on multiple components. Additionally, fiber optic probes can enable spectroscopy-based *in-situ* monitoring of neo-tissue growth during the TE process (Drieschner et al., 2020; Kandel et al., 2020). Previous studies have shown that NIRS can assess ECM in TEC, but the cell content has not been accounted for (Kandel et al., 2020; Querido et al., 2021; Yousefi et al., 2018). While visible spectroscopy has been used for cell culture characterization (Drieschner et al., 2020), it has not been studied yet for TEC characterization.

This study investigates, for the first time, the combination of visible and NIR spectroscopy for non-destructive assessment of TEC maturity through simultaneous prediction of their GAG and DNA contents. TEC constructs were prepared using two hydrogels and three growth factor protocols, providing six different construct types. The constructs were cultured for 7 or 28 days to provide two relative maturity levels for each type: immature and more mature. The NIR and Visible-Short-NIR (VIS-SNIR) spectra of the constructs were then collected simultaneously, followed by biochemical quantification of their GAGs and DNA contents. We then determined the optimal spectral range, preprocessing methods and ML algorithms for predicting the DNA and GAG content of the constructs using their NIR and VIS-SNIR spectra. For each biomarker, the performance of the top-performing VIS-SNIR and NIR-based models were then compared. Finally, we evaluated the accuracy of tissue maturity predictions using the GAG and DNA contents predicted by the top-performing models. We hypothesized that changes in DNA and GAG contents of TEC constructs during tissue growth are associated with changes in the visible and NIR spectra of the constructs.

## 2 Material and methods

### 2.1 Samples preparation

**Fig. 1:**
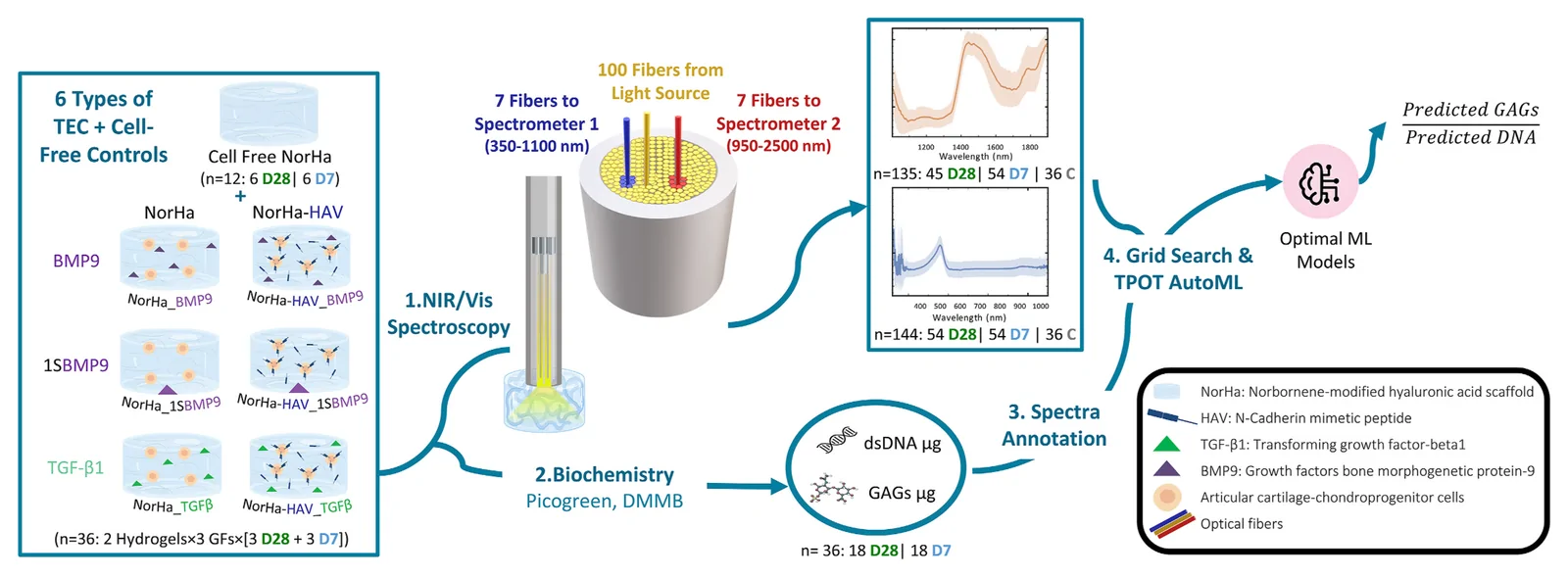
A schematic representation of the experimental approach of the study. TEC: Tissue-Engineered Cartilage; D28 & D7: constructs cultured for 28 and 7 days (i. e., relatively mature and immature), respectively; 1SBMP9: BMP9 added as a single shot at the beginning of the culture; BMP9: continuous BMP9 supplementation during culture; DMMB: Dimethylmethylene Blue Assay, GAGs: Glycosaminoglycans; ML: Machine Learning. Some elements of the figure were created using Bio-Render. (For interpretation of the references to colour in this figure legend, the reader is referred to the Web version of this article.)

Six types of mature (*n* = 6 × 3) and immature (*n* = 6 × 3) TEC samples were included in this study (Fig. 1). These samples were prepared using articular cartilage-derived chondroprogenitor cells (Supplementary Materials) as part of a previous study (Abinzano, 2024; Elkadi et al., 2024). The cells were expanded to passage 3 in chondroprogenitor expansion media, and then 20 million cells/mL were encapsulated in two types of hydrogels. One type of those hydrogels was norbornene-modified hyaluronic acid (NorHa), while the other was NorHa with conjugation of an N-cadherin mimetic peptide (NorHa--HAV) (Abinzano, 2024; Levato et al., 2017).

The constructs (n = 36) were then incubated in chondrogenic media supplemented with either 10 ng/mL transforming growth factor-beta1 (TGFβ1) and 100 nM dexamethasone (*n* = 12) or 100 ng/mL bone morphogenetic protein-9 (BMP9; n = 24). BMP9 was added as a single shot at the beginning of the culture (1SBMP9; n = 12) or with every media change (BMP9; *n* = 12) (Abinzano, 2024; Abinzano et al., n.d.). These different growth factor treatments provided three further sub-types for each of the two types of hydrogels (a total of six types of samples (n = 6 × 6); Fig. 1).

The constructs were incubated for either 7 or 28 days at 37 °C, 5 % CO_2,_ and a relative humidity of 95 % to provide relatively immature (*n* = 18) and mature (*n* = 18) cartilage constructs, respectively. Cell-free controls (*n* = 12) were also prepared using the same protocol but without cells and incubated for 7 days (*n* = 6) and 28 days (*n* = 6). All harvested constructs were washed with PBS and then frozen in 250 μl of PBS until spectroscopy (Abinzano, 2024). Both expansion and chondrogenic media were refreshed twice a week. The constructs and media preparation are further detailed in the Supplementary Materials.

### 2.2 Spectroscopic measurements

The spectra in the VIS-SNIR (350–1100 nm) and NIR (950–2500 nm) ranges were acquired simultaneously three times from each sample (a total of 144 spectra) using a custom diffuse reflectance fiber optic probe connected to a multichannel spectrometer (AvaSpec Multichannel spectrometer, Avantes BV, The Netherlands). The probe has an outer diameter of 3.25 mm and a tip window of 2 mm diameter and holds 114 optical fibers (diameter =100 μm). These include 100 fibers emitting light (total power =16 mW) from a halogen light source (Avalight-HAL-S, Avantes BV), seven fibers collecting light to a spectrometer covering the VIS-SNIR range with a resolution of 0.6 nm (AvaSpec-ULS2048L, Avantes BV), and seven fibers collecting light to a spectrometer covering the NIR range with a resolution of 6.53 nm (AvaSpec-NIR256–2.5, Avantes BV). The spectra were acquired as averages of 100 scans with an integration time of 1.1 ms and 20 ms for the VIS-SNIR and NIR spectra, respectively. The probe tip was placed in contact with and perpendicular to the center of the construct surface during the measurement (Fig. 1). The spectra were collected in absorbance mode using Avasoft software (version 8.7.0, Avantes BV), with Spectralon reflectance standard – SRS-99 (Labsphere Inc., North Sutton, USA) used as reference.

### 2.3 Biochemical analysis

The DNA and GAG contents of the constructs were then measured as previously described (Levato et al., 2017). Briefly, all samples were freeze-dried and digested overnight at 60 °C in 250 μg/mL papain, 0.01 M cysteine, 0.01 M EDTA, and 0.2 M NaH_2_PO_4_. Quant-iT PicoGreen dsDNA kit (Life Technologies, The Netherlands) was used to determine the DNA content of the constructs from the papain digests as instructed by the manufacturer. Dimethylmethylene blue assay (DMMB, Sigma-Aldrich, The Netherlands) was then used to determine the GAG content based on the protocol of Farndale et al. (1982). In short, samples were diluted and mixed with twice the volume of DMMB solution. The ratio of the absorbance at 525 nm and 595 nm of the mixture was then determined using a spectrophotometer (BioRad, Hercules, CA), and the GAG content was calculated by comparing it to known dilutions of chondroitin sulfate as standards (Farndale et al., 1982; Levato et al., 2017).

### 2.4 Data preprocessing and machine learning

**Fig. 2:**
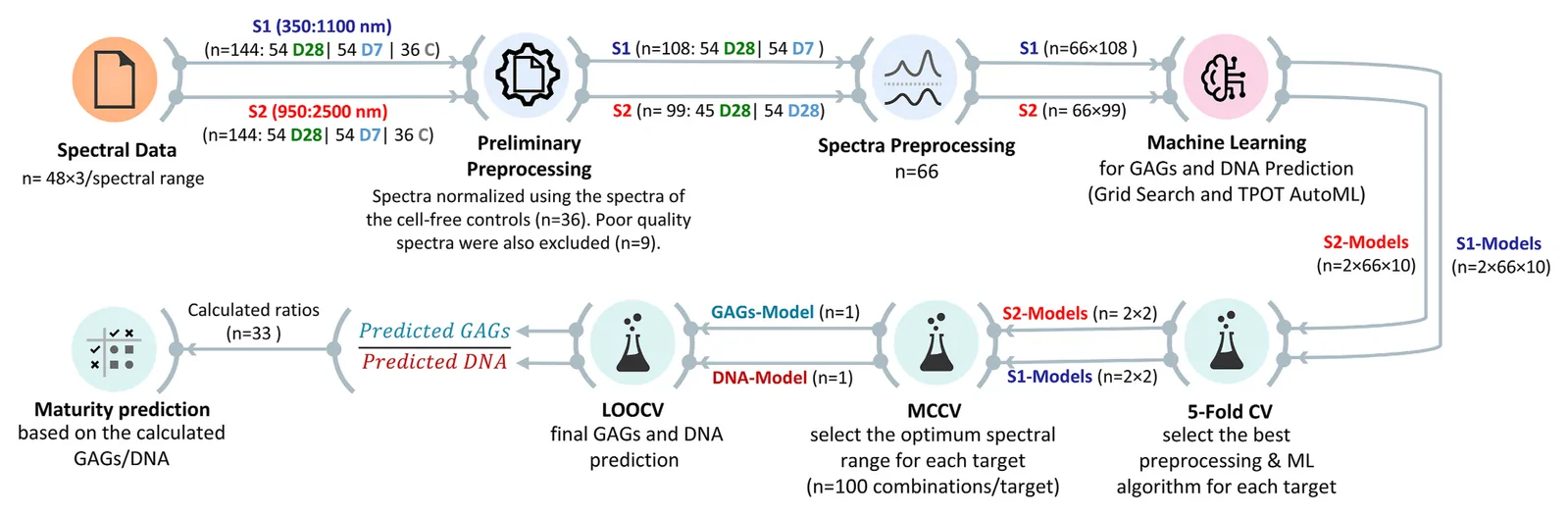
An overview of the data analysis and machine learning pipeline used in this study. S1 & S2: Spectral data from spectrometers 1 (350–1100 nm) and 2 (950–2500 nm), respectively; CV: Cross-validation; ML: Machine Learning; GAGs: Glycosaminoglycans; LOOCV: Leave-one-sample-out Cross-validation.

Preliminary spectral data preprocessing and ML analysis were conducted in Quasar 1.7 (Bioinformatics Lab, University of Ljubljana, Slovenia) to optimize the data and the analysis pipeline before further computationally intensive analysis (Toplak et al., 2021). For both the GAGs and DNA prediction, the optimal spectral ranges, ML algorithms, and their hyperparameters (including spectral preprocessing methods) were then determined using scikit-learn grid search and the AutoML platform tree-based automated machine learning (TPOT) (Le et al., 2020; Pedregosa et al., 2011). GAG and DNA contents predicted by the optimal models (based on their respective optimum spectral range, preprocessing methods, and ML algorithms) were used to calculate the GAGs/DNA ratio to predict the maturity of the constructs. An overview of this ML workflow is shown in Fig. 2.

#### 2.4.1 Preliminary models

The performances of nine ML algorithms in predicting DNA and GAG contents using their VIS-SNIR and NIR spectra were evaluated by 5-fold cross-validation (Fig. 2). Spectra with poor quality (exhibiting excessive noise and/or saturation) were excluded from the analysis. All spectra were normalized by extended multiplicative signal correction (EMSC) using the mean spectra of their corresponding cell-free controls (based on incubation duration: 7 days vs 28 days) as references. The ML algorithms and their corresponding hyperparameters are shown in Supplementary Table S1.

#### 2.4.2 Determining optimal preprocessing methods and ML algorithms for DNA and GAG content predictions

For both NIR and VIS-SNIR spectral sets, Scikit-learn’s grid search and the AutoML platform TPOT were used to determine the optimal ML algorithms (and their hyperparameters) along with the corresponding spectral preprocessing (Fig. 2). The EMSC-normalized spectra were preprocessed using 66 combinations of standard spectral preprocessing techniques (detailed in Supplementary Table S2 and Fig. S1) using the R package spec4ML (Elkadi, 2024; Morais et al., 2020). After averaging the technical replicates, each of the 66 sets of preprocessed NIR and VIS-SNIR spectra was evaluated using grid search and TPOT to identify the optimal preprocessing-algorithm combination for each spectral range, minimizing the prediction error for each biomarker. For computational efficiency and fair comparisons—especially for ML algorithms that are inefficient with high-dimensional data and for TPOT AutoML—the VIS-SNIR spectra were also binned by a reduction factor of 6, reducing the number of wavenumbers from 1356 (intervals of ~0.55) to 226 (intervals of ~3.3).

Scikit-learn grid search was performed by exploring the same nine algorithms of the preliminary models but with a larger hyperparameter search space (Table S3) for each preprocessing method. The TPOT AutoML analysis was conducted in Python in two steps for computational considerations. The first step included all 66 preprocessed spectra with AutoML search limited to 5 min. The second step was conducted using spectra from the top 3 preprocessing pipelines that showed the best performance (lowest error) in the first step, but the AutoML search was limited to 30 min or terminated upon convergence. The models were evaluated using 5-fold cross-validation. Grid search and AutoML were conducted on a supercomputer with 8 cores and ~32 gigabytes of memory.

#### 2.4.3 Determining the optimal spectral range for predicting DNA and GAG contents and the final prediction of GAGs/DNA ratios

To determine the optimal spectral range, the performance differences between the top-performing models from both spectral ranges were evaluated using Monte Carlo Cross-Validation (MCCV). MCCV was conducted by comparing the performance of the selected ML models on the same combination of 100 training and testing sets of the spectra, ensuring comparability. Each spectrum was preprocessed using the corresponding pipeline, identified in Section 2.4.2 as optimal for its spectral range and the ML algorithm employed (Fig. 2).

Leave-one-sample-out cross-validation (LOOCV) was then conducted using the preprocessed spectra of the optimal range for each of the biomarkers and their corresponding optimal ML algorithm. LOOCV was conducted by predicting the biomarkers (GAGs or DNA according to the spectral range) from the spectra of each sample (*n* = 3) using a model trained on the remaining spectra. The averages of the three predictions of each sample were compared to the actual values and used to calculate the GAGs/DNA ratio for each sample. For each sample, predictions with differences from the median of the three predictions exceeding 5 times the median absolute difference were identified as outliers and excluded. Only samples that have both NIR and VIS-SNIR spectra were included in the MCCV and LOOCV.

#### 2.4.4 Prediction of tissue maturity

To use the calculated GAGs/DNA ratios for predicting the relative tissue maturity, a cutoff value was determined as the midpoint between their minimum value in the relatively mature constructs (cultured for 28 days) and their maximum value in the immature constructs (cultured for 7 days). Based on this cutoff, the constructs were predicted as mature/ immature using their GAGs/DNA ratios calculated from the GAGs and DNA values predicted by the spectra-based ML models.

#### 2.4.5 Performance metrics and statistical analyses

Three-way ANOVA was conducted using GraphPad Prism 8.0 (GraphPad Software, USA), including multiple comparisons using a post-hoc Bonferroni test to determine whether there are statistical differences in the GAG and DNA contents (and their ratios) among the samples according to the hydrogels, growth factors, and culture time. The performance of the ML models and the correlation between the predicted and actual GAG/DNA ratios were assessed using the following metrics: Root Mean Squared Error, Mean Absolute Error (MAE), Normalized Mean Absolute Error (NMAE, calculated as 100 × MAE/ Range), and the Coefficient of Determination (*R*^2^). MAE was the primary metric for evaluation, where lower MAE means better performance. For the MCCV, a paired *t*-test was used to test the significance of the differences between the MAE of the 100 NIR spectra-based models and their corresponding VIS-SNIR-based models. All *p*-values are presented as two-sided and as corrected *p*-values for ANOVA post-hoc multiple comparisons. The classification of the constructs according to their maturity was evaluated in terms of overall accuracy.

## 3 Results and discussion

### 3.1 Constructs biochemistry

**Fig. 3:**
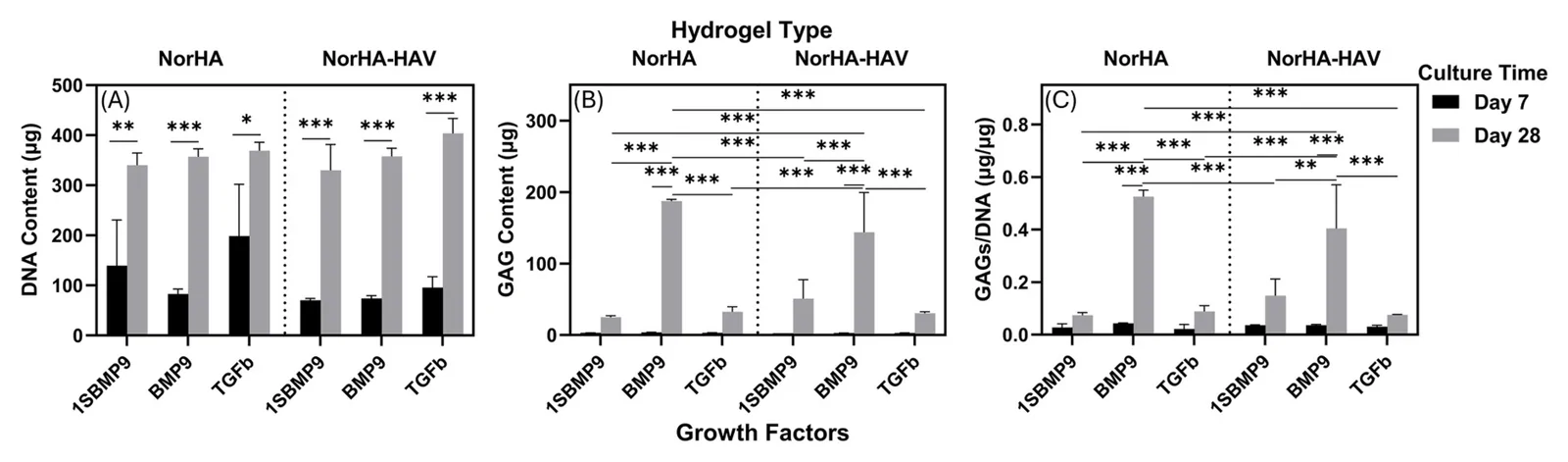
The mean ± standard deviation (SD) of the DNA (A) and GAG (B) contents of the constructs (*n* = 6 types × 3 replicates) and their corresponding GAGs normalized to DNA (C) after 7 and 28 days of culture. 1SBMP9: BMP9 added as a single shot at the beginning of the culture; BMP9 and TGFb: continuous BMP9 and TGFβ1 supplementation, respectively, during the culture period. Summary of the data and additional statistics are listed in Supplementary Table S4. Adapted from Abinzano et al.(n.d.). *p < 0.01; **p < 0.001; ***p ≤ 0.0001.

The six types of constructs showed variability in their GAG and DNA contents (Fig. 3 and Supplementary Table S4). Variations in culture time and growth factors were responsible for these inter-construct-type variations. All constructs cultured for 28 days showed higher DNA and GAG contents and GAGs/DNA ratios compared to their corresponding constructs cultured for 7 days. These increases reflect an increase in cell numbers, ECM deposition, and construct maturity. The increase in DNA with culture time was statistically significant for the six types of constructs (Fig. 3A). However, the increases in the GAG content and GAGs/ DNA ratio were only statistically significant in the two types that included continuous BMP9 supplementation (Fig. 3B and C). The limited statistical significance in the remaining four types (despite a profound increase) can be attributed to the intra-construct-type variabilities (GAGs/DNA median relative standard deviation: 15.96 %, reaching up to 78.5 %; Fig. 3C and Supplementary Table S4), which adds to the variability among the samples included in this study. There was no significant difference between the two hydrogels in GAG or DNA contents, whereas the constructs continuously cultured with BMP9 showed significantly higher GAG content and GAG/DNA ratio than the other constructs (Fig. 3).

However, in a previous study, we demonstrated that changes in growth factors and hydrogels (unrelated to the neo-tissue) can influence construct spectra (Elkadi et al., 2024). Therefore, even constructs with no significant differences in GAG or DNA contents can contribute to variations in the training data due to spectral differences associated with changes in these components. This variability among closely related samples is advantageous for training robust spectra-based ML models, as it enables the identification of wavelengths (features) that are conserved despite such variations. Such robust models would be more capable of handling real-world data variabilities (Elkadi et al., 2021, 2024).

### 3.2 Raw spectra and preliminary preprocessing

The mean spectra before and after preliminary preprocessing are shown in Supplementary Fig. S2. Both spectral ranges show differences between the immature and the more mature constructs (cultured for 7 and 28 days, respectively). However, they also show differences between the cell-free controls incubated for 7 and those incubated for 28 days under the same conditions (Fig. S2A and S2B). Since spectral data were acquired in the same measurement session by the same operator, these changes can be attributed solely to hydrogel alterations. These alterations may be due to differences in exposure time to the tissue culture conditions. For instance, any uncross-linked hydrogel can be washed off during media changes, or the hydrogels might be degrading over time. They could also be attributed to the different freezing times due to the differing storage durations between the samples grown for 7 and 28 days. Because these changes are not related to the growing tissue, EMSC has been used to correct for spectral changes related to the hydrogels in the constructs.

EMSC is a model-based preprocessing method commonly employed in vibrational spectroscopy to correct for spectral changes attributed to phenomena other than the chemical components of interest (Afseth and Kohler, 2012). By using the average spectra of time-matched cell-free controls as reference spectra for EMSC correction, the algorithm corrects for spectral variations common to both the constructs and their corresponding cell-free controls. Moreover, EMSC corrects for other physical effects such as path length variations and scattering effects (Afseth and Kohler, 2012).

However, the NIR spectra exhibited significant noise, necessitating further preprocessing. Although preprocessing methods can handle substantial noises in the spectra without significant loss of information (Manley, 2014), the NIR spectra of three samples were excluded as they exhibited saturation at most wavelengths above 1400 nm. Absorbances at wavelengths above 1900 nm were also excluded from the analysis as they exhibited significant saturation in most samples (Fig. S2B and S2D). This poor quality of the NIR spectra can be attributed to the challenge of optimizing the setup for simultaneous spectral acquisition using both spectrometers; achieving optimal quality for the VIS-SNIR spectra compromised the quality of the NIR spectra. Thus, further studies are required to optimize the setup for simultaneous spectral acquisition to improve spectral quality. Alternatively, sequential acquisition can be considered, allowing each method to use its optimal setup. As previous NIRS-focused studies employed non-contact measurements of engineered cartilages (Kandel et al., 2020), setup optimization should also address the probe-sample distance for each spectral range while considering potential interference from culture media during continuous monitoring—especially if the probe is not in direct contact with the sample.

### 3.3 Preliminary models

The performances of the top-performing preliminary models for each spectral range are presented in Supplementary Table S5. Normalizing the spectra of the constructs to the spectra of their corresponding cell-free controls using EMSC was essential to obtain ML models with adequate performance. Models trained with spectral data without this normalization showed lower performance than those trained with normalized spectra (Table S5). This lower performance can be attributed to changes in the spectra associated with changes in the hydrogels that can negatively influence the performance of the ML models in predicting tissue-related changes (Elkadi et al., 2024).

In the preliminary models based on the normalized data, the VIS-SNIR spectra required a relatively simple algorithm, a decision tree, to achieve the top-performing models for predicting both GAG and DNA contents, with NMAE of 8.92 % and 9.51 %, respectively. Conversely, the NIR spectra required a more advanced algorithm, AdaBoost, to achieve the top-performing models for predicting GAG and DNA contents, with NMAE of 7.21 % and 10.51 %, respectively (Table S5). This reflects the relative complexity of the NIR spectra compared to the VIS-SNIR spectra, which could be due to inherent properties of the spectra, the slightly lower number of NIR spectra used (after excluding the three samples with poor quality), or the relatively lower quality of the NIR spectra that require additional preprocessing.

### 3.4 Optimal preprocessing methods and ML algorithms for predicting DNA and GAG contents

In chemometrics, the optimal spectral preprocessing method is typically determined through trial and error (Manley, 2014; Mohamed et al., 2015). A similar strategy is employed in ML, where different algorithms and their corresponding hyperparameters—selected manually via grid search or automated through AutoML—are evaluated on the same dataset to identify the configuration that minimizes prediction error (Le et al., 2020; Pedregosa et al., 2011). Accordingly, in this study, grid search and TPOT AutoML were applied in combination with various spectral preprocessing pipelines to identify the preprocessing-algorithm combination with the lowest MAE for predicting DNA and GAG contents for each spectral range.

No specific preprocessing nor a specific ML algorithm consistently yielded the highest performance in predicting the biomarkers (Fig. S3), which is consistent with the No Free Lunch theorem, where no optimization algorithm is universally superior to all others across all possible problems (Wolpert and Macready, 1997). Thus, in such chemometric problems, for each biomarker, a specific ML algorithm will provide the most accurate prediction, and this algorithm requires an optimal spectral range and preprocessing to achieve optimal spectral feature utilization. Thus, searching for the optimal combination of preprocessing method and ML algorithm was essential for identifying the best model.

The grid search and TPOT AutoML showed that higher performance can be achieved by advanced ML algorithms for the spectral data from both ranges after specific further preprocessing in addition to the EMSC normalization applied to the spectra. The NMAE in predicting GAG and DNA contents were as low as 4.5 % (for a VIS-SNIR-based model) and 5.6 % (for a NIR-based Model), respectively (Fig. S3).

### 3.5 Optimal spectral range for predicting DNA and GAG contents and final prediction of the GAGs/DNA ratios

Given the limited sample size of this study, MCCV was employed to compare the MAE of the top-performing models of the two spectral ranges across the same 100 randomly selected Train/Test sets. Then, for each biomarker, the model that showed the lowest MAE on average during MCCV was further evaluated using LOOCV (replicates included) to get predictions of GAG and DNA contents for each of the 33 samples. These predictions were used to calculate the GAG/DNA ratios and compared with their actual values calculated by the destructive biochemical reference methods.

**Fig. 4:**
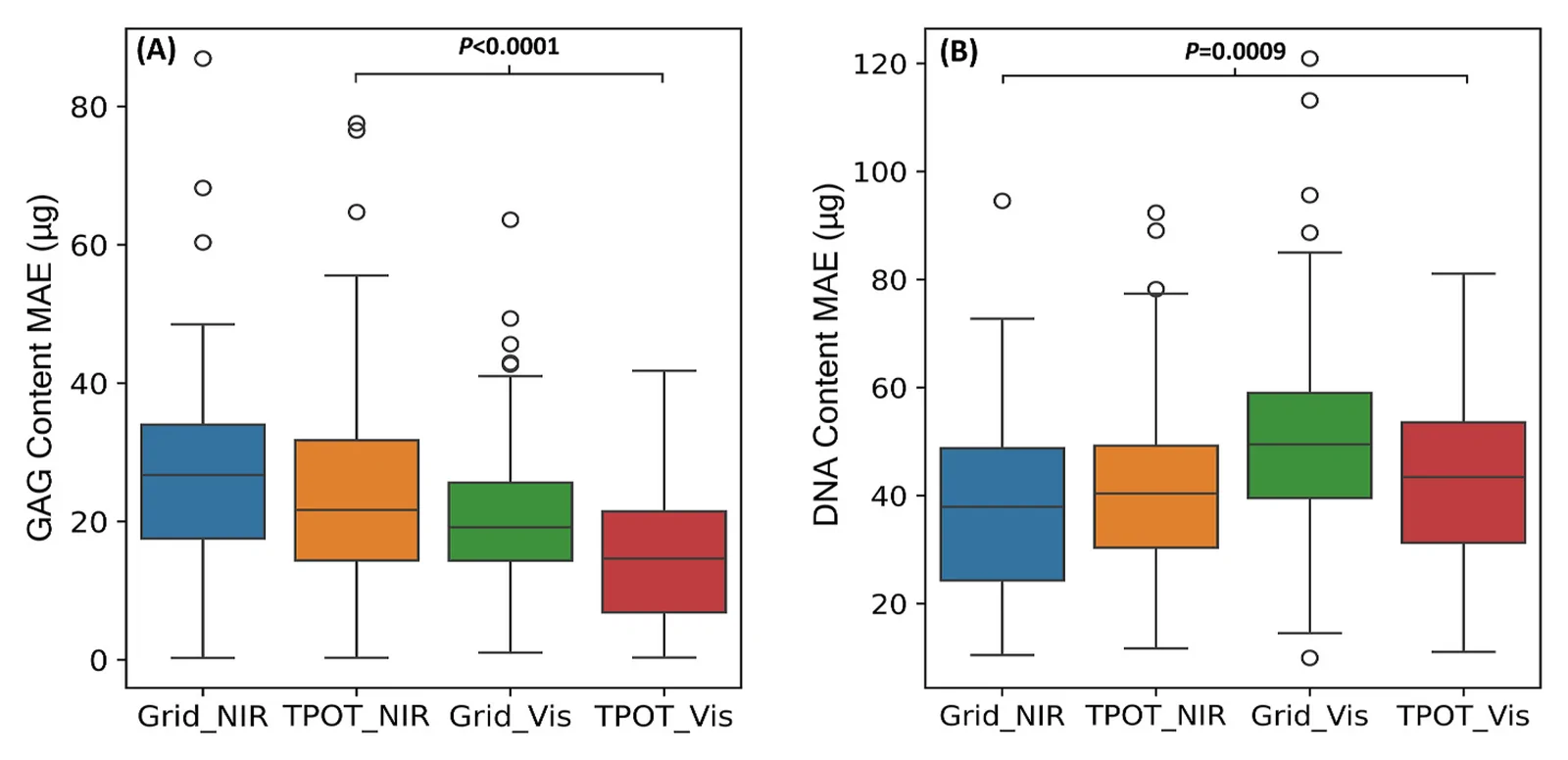
Box plot of the mean absolute error (MAE) of the models selected by grid search and TPOT in predicting the GAG (A) and DNA (B) contents using the optimally preprocessed NIR (950–1900 nm) and Visible-Short-NIR (350–1100 nm) spectra across the 100 train/test combinations randomly selected during the Monte Carlo Cross-Validation. GridNIR: models selected by the grid search using the NIR spectra; TPOTNIR: models selected by the TPOT AutoML using the NIR spectra; GridVis: models selected by the grid search using the Visible-Short-NIR spectra; TPOTVis: models selected by the TPOT AutoML using the Visible-Short-NIR spectra. Supplementary Fig. S4 shows a heat map of the errors for the 100 Train/Test combinations. Summary of the data and additional statistics are listed in Supplementary Table S6.

**Fig. 5:**
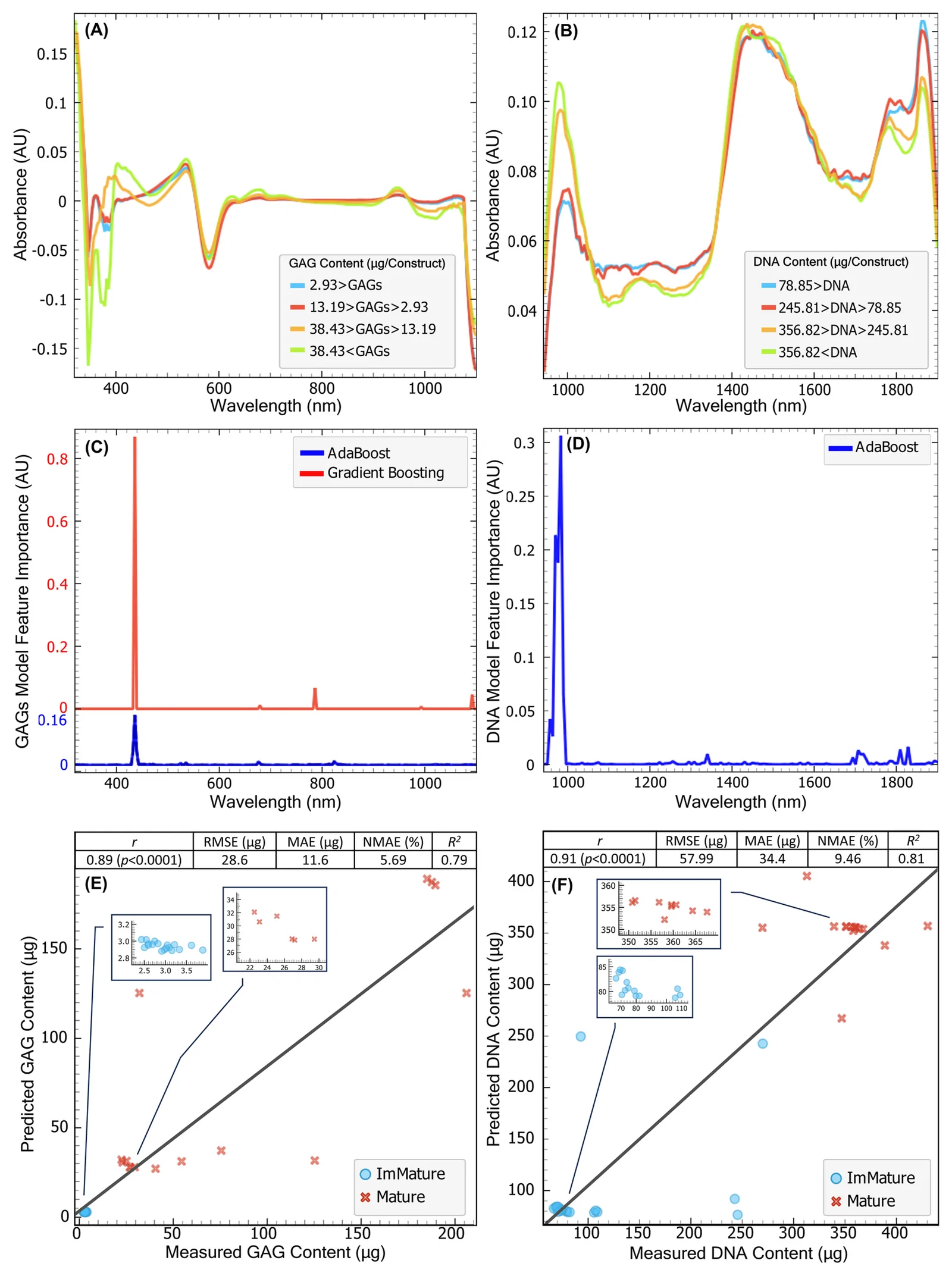
The mean spectra of the constructs after the employed preprocessing: (A) First Derivative of the Visible-Short-NIR spectra for GAG content prediction; (B) NIR spectra smoothed and normalized by unit vector normalization for the DNA content prediction. Feature importance of the stacked models for GAG content prediction (C) and the model for DNA content prediction (D). Performances of the optimized models in predicting GAG content (E) and DNA content (F) in leave-one-sample-out cross-validation. *r*: Pearson Coefficient; RMSE: Root Mean Square Error; MAE: Mean Absolute Error; NMAE: Normalized Mean Absolute; *R*^*2*^: Coefficient of Determination; AU: Arbitrary Unit.

The top-performing VIS-SNIR-based model (selected by TPOT; TPOTVis) showed significantly lower MAE in predicting GAG content compared to the top-performing NIR-based model (selected by TPOT; TPOTNIR) with an average difference of 8.3 μg (*p*= 2.12E-08), despite considerable performance variations across the 100 MCCV sets (Fig. 4 and Fig. S4 and Supplementary Table S6). This better performance of the VIS-SNIR-based model could be attributed to the stronger interaction (mainly scattering) of visible light with the ECM represented by the GAG content (Kafian-Attari et al., 2023). The selected TPOT pipeline included robust scaling, followed by training of an AdaBoost Regressor, using gradient boosting regressor as a middle estimator (Fig. S5), and achieved its optimal performance using the first derivative (window length = 15) of the EMSC-normalized VIS-SNIR spectra (Fig. 5A). This model showed a satisfactory prediction of the GAG content during the LOOCV, with an *R*^2^ of 0.79 (Fig. 5E).

Conversely, the top-performing NIR-based model (selected by the grid search; GridNIR) showed significantly lower MAE compared to the top-performing VIS-SNIR-based model (selected by TPOT; TPOTVis) in predicting DNA content with an average difference of 5.69 μg (*p* = 0.0009; Fig. 4 and Fig. S4, and Supplementary Table S6). This better performance of the NIR-based model could be attributed to the higher penetration depth of NIR light and its lower scattering by the ECM components (Kafian-Attari et al., 2023). The grid-search-selected model is an AdaBoost Regressor with a learning rate of 0.01 and 100 estimators, trained on EMSC-normalized NIR spectra further preprocessed with Savitzky-Golay smoothing (window length = 11) followed by unit vector normalization (Fig. 5B). This model also showed satisfactory performance in predicting DNA content during LOOCV with an *R*^2^ of 0.81 (Fig. 5F).

The feature-importance analysis of the models shows that they are mainly influenced by spectral regions with pronounced changes associated with the level of the biomarkers; contents, especially for the bands around 435 nm and 983 nm for GAGs and DNA, respectively (Fig. 5). Although the models were trained on the GAG and DNA contents of the constructs, it is worth noting that these biomarkers are surrogates for the ECM and cellular content of the constructs, respectively. Similarly, the models could be influenced by scattering effects more effectively caused by other ECM components, such as the larger collagen macromolecules, and by cells, rather than directly measuring the GAGs and DNA (Drieschner et al., 2020; Kafian-Attari et al., 2023). Therefore, the absorbance around 983 nm can be attributed to the third overtone of the vibration of the C-H bonds (Badr Eldin, 2011), which is abundant in cellular components like the cell membrane. Meanwhile, the absorbance around 435 nm can be attributed to phenol red—a pH indicator in the culture media (Morgan et al., 2019)—which can act as a negative stain by being contained in the construct during early swelling of the hydrogel and then replaced by the ECM as the construct matures.

**Fig. 6:**
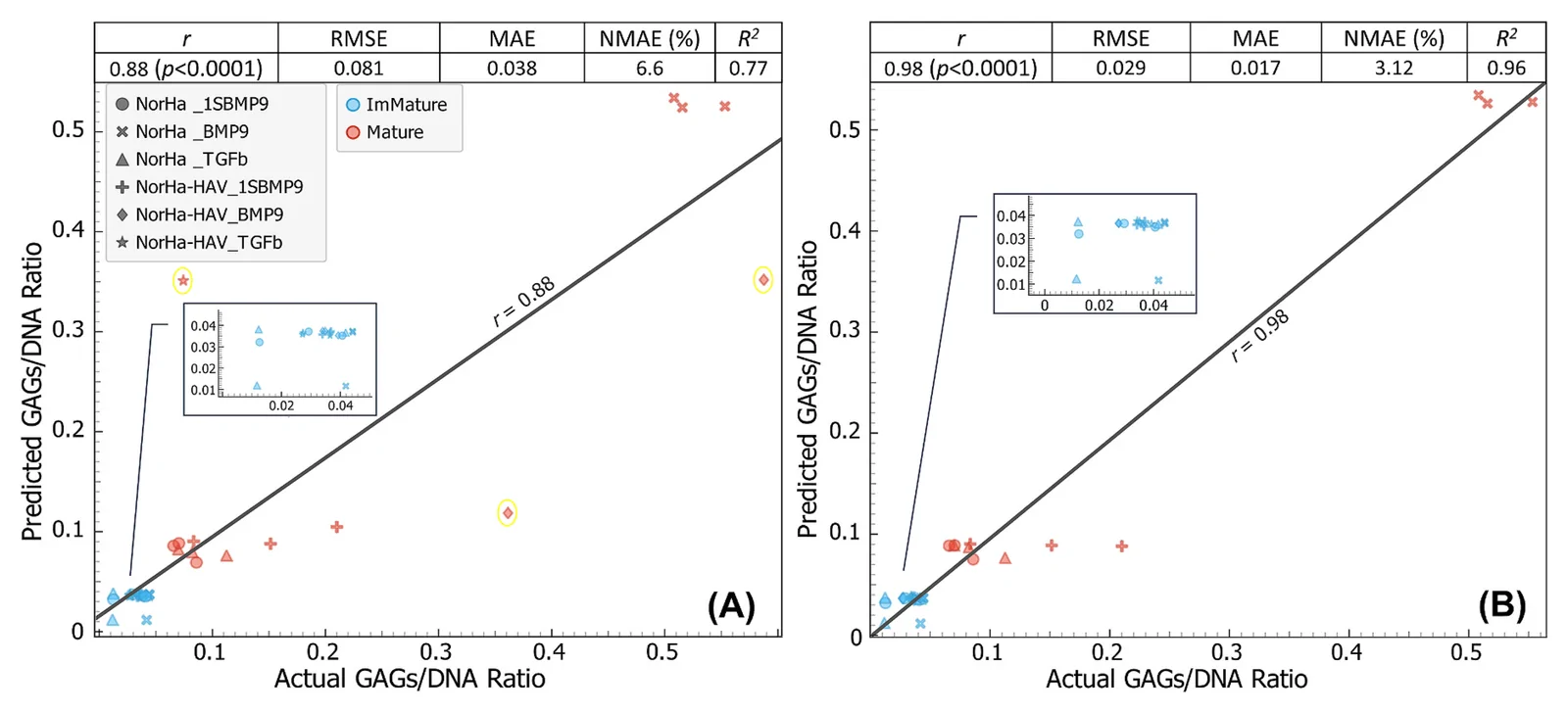
Predicted GAG/DNA content ratio vs. the actual GAG/DNA content ratio using all samples (A) and after excluding the spectra of the samples with limited replicates from testing (B): matured samples of NorHa-HAV with continuous BMP9 supplementation (NorHa-HAV_BMP9) and matured samples of NorHa-HAV with continuous TGFβ1 supplementation (NorHa-HAV_TGFb). *r*: Pearson Coefficient; RMSE: Root Mean Square Error; MAE: Mean Absolute Error; NMAE: Normalized Mean Absolute Error; *R*^*2*^: Coefficient of Determination.

The finally predicted GAGs/DNA ratios showed adequate correlation with the measured ratios (*R*^*2*^ = 0.77, Fig. 6A). This performance is comparable to other more complex spectroscopic methods reported in previous studies. In a previous study, a Raman-based model predicted the GAGs/DNA ratios of cartilages engineered from nasal chondrocytes with *R*^*2*^ of 0.78 (Power et al., 2020). In another study, a fiber-optic multimodal system based on fluorescence lifetime imaging and ultra-sound backscatter microscopy predicted collagen/DNA ratios of self-assembled TE articular cartilage with *R*^*2*^ of 0.73 (Haudenschild et al., 2022). However, the models in these studies were tested on smaller sample sizes (6 and 18 samples, respectively) of less diverse constructs. The Raman spectroscopy also required fixation on CaF2 slides. Fiber-optic Raman spectroscopy, however, has shown potential for non-invasive ECM quantification (Bergholt et al., 2017; Haudenschild et al., 2022; Power et al., 2020). Further studies are required to compare the relatively simpler NIR/Visible spectroscopy method proposed in the present study with these more complex fiber-optic spectroscopic methods on the same set of samples. Integrating these spectroscopic methods into a potentially more accurate fiber-optic-based multimodal method should also be considered in future studies.

Further investigation of the samples with high prediction errors revealed that the model performance was negatively affected by two types: mature NorHa-HAV_TGFb and mature NorHa-HAV_BMP9. Excluding these samples (also from model development) improved the correlation between the actual and the predicted GAG/DNA ratios (*R*^2^ improved to 0.96) (Fig. 6). This effect can be attributed to the limited number of spectra from these sample types included in the model, as samples from these types had been excluded due to the poor quality of their NIR spectra. Thus, only one mature NorHa-HAV_TGFb and two mature NorHa-HAV_BMP9 samples were used for model development and evaluation. During LOOCV, the model had only one or none of these sample types in the training set when testing samples from these types. Since changes in the construct’s non-neotissue components influence the spectra of the samples and the performance of spectral-based ML models (Elkadi et al., 2024), the model’s predictive ability for these specific samples was limited by the insufficient training data available to account for their construct-specific spectral differences. This underscores that the model’s predictive ability is limited to the construct types included in its training. Thus, for the maturity of a construct to be reliably predicted, spectra of representative samples from the construct type must be included in the training of the model.

### 3.6 Prediction of tissue maturity

The minimum actual GAGs/DNA ratio observed in mature constructs was 0.06573, while the maximum ratio in immature constructs was 0.04423. Therefore, a threshold of 0.055 was selected as the cutoff for classifying the constructs as relatively mature (high GAGs/DNA) and immature (low GAGs/DNA). The GAGs/DNA ratios predicted by the VIS-SNIR/NIR-based models achieved a 100 % accuracy (33/33) in classifying the constructs according to their maturity, even for samples whose GAG and DNA contents were poorly predicted by the models (confusion matrix is shown in Fig. S6).

In this study, the maturity of constructs was defined based on the culture duration, with constructs cultured for 28 days assumed to be relatively more mature than the corresponding constructs cultured for 7 days. This assumption is supported by the overall higher GAGs/DNA ratios observed in constructs cultured for 28 days compared to those cultured for 7 days (Fig. 3). However, this study included samples harvested after only two culture durations with distinct differences in their biochemical composition and, consequently, their spectra (Figs. 3, 5 and 6). These distinct differences can be the reason for the perfect performance of the predicted GAGs/DNA ratios in classifying the samples according to their relative maturity. Further studies that include less biochemically distinct samples, i.e., harvested at closer time points, are required to evaluate the robustness of this approach in addressing real-world TE challenges.

## 4 Conclusion

This study presents a proof of concept for a novel multimodal approach that uses visible and NIR spectroscopy to assess the maturity of TEC constructs by predicting their GAG and DNA contents. ML models based on VIS-SNIR and NIR spectra of the constructs demonstrated accurate predictions of these biomarkers, which are important for evaluating construct maturity. Optimal prediction of GAG content was achieved using VIS-SNIR spectra, while DNA content was best predicted with NIR spectra. This study is the first to demonstrate that an ML model trained on spectral data from different types of tissue-engineered constructs can effectively predict their biochemical composition. However, this approach would require retraining the model with spectra of any new construct types. It would also require collecting the spectra of cell-free controls for spectral preprocessing. Additionally, due to the limited sample size for each construct type and culture duration, further studies are needed to evaluate the performance of visible and NIR spectroscopy in assessing more complex samples and to validate the approach with independent test sets.

Such non-destructive fiber-optic-based methods could enable continuous TE process monitoring, thus enabling optimization of TE strategies comparisons and TEC constructs maturity evaluation for cartilage repair. By tracking the GAG/DNA levels, constructs could be harvested upon reaching a prespecified threshold rather than relying on fixed culture durations, which often result in inconsistent maturity. This approach would also overcome inconsistencies in time-to-maturity due to donor-to-donor variability, ensuring reproducible quality of TEC for clinical use and enabling a more personalized process. It could also improve research efficiency by enabling comparisons based on the time required to reach a target GAG/DNA level rather than the level achieved after a fixed culture period, saving time and resources. Additionally, continuous monitoring would enable real-time detection of unexpected growth patterns—such as plateaus, fluctuations, or post-peak declines—that might be missed with endpoint or intermittent measurements. Fiber optics can also facilitate real-time multimodal spectroscopic analysis of the construct, potentially enhancing evaluation accuracy by providing complementary spectral data that offers more comprehensive biochemical insights. However, the optimal setup for simultaneous or sequential spectral acquisition should be determined to ensure high-quality spectra for each spectroscopic method. Integrating this fiber-optic-based approach into bioreactors could also allow efficient control of the culture parameters using real-time information about the cultured constructs.

## Supplementary Material

Supplementary data to this article can be found online at https://doi.org/10.1016/j.bios.2025.117587.

## Data Availability

Data will be made available on request.
